# The Physical Exploration of the Rectum, with an Appendix on the Ligation of Hæmorrhoidal Tumors

**Published:** 1870-11

**Authors:** 


### Books Review.

---

*The Physical Exploration of the Rectum, with an appendix on the Ligation of Hæmorrhoidal Tumors.* By WILLIAM BODENHAMER, A.M., M. D. WILLIAM WOOD & Co., N. Y. 1870.

The author's introductory shows the frequency and importance of Rectal disease, and that this *region* is susceptible of exact observation, and its diseases of scientific analysis, of safe, certain and appropriate treatment. He speaks of the general neglect of diseases of the rectum, and quotes a writer in the *Medical Chirurgicat Review*, who observed that "*bey,ond the treatment of Fistula in Ano and Hæmorrhoids, the surgery of the rectum is a sort of land of the Cimmerians, where quacks alone can breathe, and where humbug darkens the air.*" Our author then introduces a very complete anatomical description of the region, and gives cuts, which well illustrates his text.

In this chapter upon Physical Exploration is the chief attraction. The points to be observed, and the manner of observing, are carefully described. All the various instruments for observing these parts are described, and the same are shown also by wood-cuts, which at once shows the manner of use. Speaking of the *Recto-Colonic Endoscope*, he says: I have thus denominated the instrument, by the use of which, and a poweıful light, the superior portion of the rectum, and inferior part of the sigmoid flexure of the colon may be accurately and minutely examined. It renders accessible to inspection a portion of the intestinal canal, a part of the iliac colon, which has heretofore been shrouded with impenetrable darkness" The appendixupon the ligation of *Hæmorrhoidal Tumors* is very instructive, and worthy of careful perusal.

Altogether this little book is much more than it pretends to be, and we advise our readers to send for it. It can be sent by mail, bought from all Medical Book Stores, and costs a great deal less than it is worth.

---

We have received the September number of the *Journal of Cutaneous Medicine and Diseases of the Skin*, which was formerly under the editorial charge of Erasmus Wilson, F.R S., until on account of his increased duties in connection with his appointment as Professor of Dermatology in the Royal College of Surgeons, compelled him to resign the publication of this Journal. We are happy to see that Dr. H. S. Purdon, of Belfast, has undertaken the continuance of the Journal, which, aided by his experience and ability, we trust will continue to occupy its former high position in the esteem of its readers.

The *London Chemist and Druggist*, published monthly. and containing thirty pages of reading matter, and as many more of advertising, is well edited, and presents a great variety of departments, and a large amount of intelligence. It is of interest to all who wish to keep posted regarding the progress of Pharmacy in Great Britain.

The *American Chemist* is the title of a monthly Journal of theoretical, analytical and technical chemistry, which succeeds a reprint of the *Chemical News*. The editors of the present publication, are Chas. F. Chandler, Ph. D., and W. H. Chandler, of the Columbia School of Mines, of whose well-known ability our readers are probably informed. We should judge from the specimen number of the Journal before us that the publication is an eminently meritorious one, and that it will be welcomed by the chemists and manufacturers of the country. It is published by Wm Baldwin & Co., of New York City.

The *Archives of Science* and transactions of the Orleans County Society of Medical Science, is a quarterly, the first number of which is issued this month, at Newport, Vt. Its editors are J. M. Currier, M. D., and Geo. A. Hinman, M. D , who state that the publication will contain sixty-four pages, and will be made up of original articles upon all scientific subjects, especially giving the results of original observations and researches respecting scientific matters of the State, although it will be open to subjects outside the State, by Vermont men. We wish the Journal much success.

The *Indiana Journal of Medicine* is a newly-started publication, edited at Indianapolis, by Drs Thad, M. Stevens, W. B. Fletcher, and Guido Bell. The general character of its articles, its editorial department and its style of publishing, are well worthy of commendation.

Part First, of a descriptive catalogue of the New Sydenham Society's Atlas of Portraits of Diseases of the Skin, has been sent us by Lindsay & Blakiston, of Philadelphia, who are authorized to receive subscriptions of membership in the Society, are prepared to furnish information respecting the Society, and are ready to furnish its valuable publications at the regular rates.

